# Low-dose exposure to bisphenols A, F and S of human primary adipocyte impacts coding and non-coding RNA profiles

**DOI:** 10.1371/journal.pone.0179583

**Published:** 2017-06-19

**Authors:** Marie Verbanck, Mickaël Canouil, Audrey Leloire, Véronique Dhennin, Xavier Coumoul, Loïc Yengo, Philippe Froguel, Odile Poulain-Godefroy

**Affiliations:** 1 University Lille, CNRS, CHU Lille, Institut Pasteur de Lille, UMR 8199—EGID, Lille, France; 2 INSERM UMR-S 1124, Toxicologie Pharmacologie et Signalisation cellulaire, Paris, France; Université Paris Descartes, ComUE Sorbonne Paris Cité, Paris, France; 3 Department of Genomics of Common Disease, School of Public Health, Imperial College London, United Kingdom; INRA, FRANCE

## Abstract

Bisphenol A (BPA) exposure has been suspected to be associated with deleterious effects on health including obesity and metabolically-linked diseases. Although bisphenols F (BPF) and S (BPS) are BPA structural analogs commonly used in many marketed products as a replacement for BPA, only sparse toxicological data are available yet. Our objective was to comprehensively characterize bisphenols gene targets in a human primary adipocyte model, in order to determine whether they may induce cellular dysfunction, using chronic exposure at two concentrations: a “low-dose” similar to the dose usually encountered in human biological fluids and a higher dose. Therefore, BPA, BPF and BPS have been added at 10 nM or 10 μM during the differentiation of human primary adipocytes from subcutaneous fat of three non-diabetic Caucasian female patients. Gene expression (mRNA/lncRNA) arrays and microRNA arrays, have been used to assess coding and non-coding RNA changes. We detected significantly deregulated mRNA/lncRNA and miRNA at low and high doses. Enrichment in “cancer” and “organismal injury and abnormalities” related pathways was found in response to the three products. Some long intergenic non-coding RNAs and small nucleolar RNAs were differentially expressed suggesting that bisphenols may also activate multiple cellular processes and epigenetic modifications. The analysis of upstream regulators of deregulated genes highlighted hormones or hormone-like chemicals suggesting that BPS and BPF can be suspected to interfere, just like BPA, with hormonal regulation and have to be considered as endocrine disruptors. All these results suggest that as BPA, its substitutes BPS and BPF should be used with the same restrictions.

## Introduction

A variety of chemicals has been found to disrupt the endocrine system of animal models, and chemical exposure has been associated with adverse developmental and reproductive effects in fish and other wildlife [1]. Especially, bisphenol A (BPA) exposure has been suspected to contribute to obesity and metabolic disorders [2].

BPA was industrially used to manufacture polycarbonate plastics and epoxy resins, which are used in baby bottles, toys and medical devices, as protective coatings on food containers, for composites and sealants in dentistry, as well as in carbonless thermal papers. The human population has a widespread exposure to BPA as it has been detected in 95% of the samples examined at concentrations ≥0.1 μg/L in urine [3]^,^[4]. Associations were shown between BPA exposure and adverse perinatal, childhood, and adult health outcomes, including reproductive and developmental effects, metabolic disease, and other health effects [5]. High urinary BPA levels were found to be associated with obesity and type 2 diabetes (T2D) in several studies [6]^,^[7]^,^[8], but not all [9].

BPA has been banned from baby’s bottles in EU and more recently in France from any food and beverage containers. New potentially toxic substitutes are emerging: there has been a gradual shift towards the use of bisphenol analogs since manufacturers have begun to remove BPA from their products. Bisphenol S (BPS) and bisphenol F (BPF) are now commonly used as substitutes (S1 Fig). BPS is used for a variety of industrial applications, for example, as a wash fastening agent in cleaning products, an electroplating solvent, and a constituent of phenolic resin. BPS is also used as a developer in thermal paper. BPF is used to make epoxy resins and coatings, especially for systems needing increased thickness and durability such as tank and pipe linings, industrial floors, road and bridge deck toppings, structural adhesives, grouts and electrical varnishes [10]. BPF was also recently demonstrated to be present in mustard which suggested a human exposure to BPF by ingestion [11]. In urine samples collected in 2009–2012 from adults in the United States, BPA was detected at the highest frequency and median concentration (95%, 0.72 ng/mL), followed by BPS (78%, 0.13 ng/mL) and BPF (55%, 0.08 ng/mL) [12]. Human exposure to BPS and BPF is expected to increase due to regulations on BPA.

The effects of BPA on estrogen receptors (ERs) have been extensively studied. Nevertheless, the binding affinity of BPA for estrogen receptors ERα and ERβ is very low and it is now clear that BPA can and does bind multiple other targets within the nucleus and on the cell membrane [13]. Based on text mining data, more than 271 proteins were reported to have a role in different biochemical processes in which BPA has been involved [14]. In addition to estrogenic action, these proteins belong to diverse biochemical pathways and, in particular, can be related to T2D and obesity physiology. Since BPA can target multiple pathways, we hypothesized that bisphenol substitutes may have a similar behavior.

Our main objective, in this study, was to evaluate genome-wide gene expression responses to the exposition of BPA, BPS and BPF. Considering the impact of BPA on obesity [15], we aimed at determining how bisphenols could impair adipocyte physiology. In order to mimic chronic bisphenol exposure, we chose to incubate bisphenols at a physiologically relevant exposure dose, also known as a “low-dose “, i.e. 10 nM. Doses below 1 × 10^−7^ M for *in vitro* BPA effects are currently considered as “low dose” and are levels below the current lowest observed effect level (LOAEL) in traditional toxicological studies [16]. In the human, BPA is absorbed from the gastrointestinal tract quickly, conjugated with glucuronic acid in the liver, and BPA–glucuronide is rapidly filtered from the blood by the kidneys and excreted in urine [17]. Taking into account quick fluctuations of serum BPA concentrations due to time and dose exposures, 10 nM is a concentration described to be within the range of concentrations usually encountered for BPA in biological fluids in a general population [18]. A higher dose (10 μM), frequently used in literature despite poorly relevant to human exposure, was also assayed to detect potential dose-specific effects. In addition, we noted that non-monotonic dose-response relationships were demonstrated for bisphenol A [19], which dissuaded us from trying to get dose-response information and using a wider range of concentrations.

We therefore compared the effects of BPA, BPS and BPF added at 10 nM and at 10 μM in the differentiation medium of primary human pre-adipocytes, on different RNA levels. We used cell cultures derived from three different patients in order to avoid overestimating effects resulting from individual susceptibility and to focus on general mechanisms. In addition, our strategy allowed the study of the broad family of RNAs: mRNA, microRNAs (miRNAs) and other non-coding RNAs in order to determine a potential role of epigenetic mechanisms in bisphenols effects. A microRNA (miRNA) is a small non-coding RNA molecule (constituted of about 22 nucleotides) that plays a role in RNA silencing and post-transcriptional regulation of gene expression [20]. Small nucleolar RNAs (snoRNAs) are a class of small RNA molecules that primarily guide chemical modifications of other RNAs, mainly ribosomal RNAs, transfer RNAs and small nuclear RNAs or may have a role in the regulation of alternative splicing [21]. Long non-coding RNAs (lncRNA) are non-protein coding transcripts longer than 200 nucleotides. They play a role in the regulation of gene transcription and also control various aspects of post-transcriptional mRNA processing [22]. These different non-coding RNA classes are detected with our set of microarrays.

Using modern hypothesis-free genome-wide technologies may contribute to make significant progress in endocrine disruptor mechanistic knowledge and open the way to unsuspected responsive pathways of interest, either for basic science or for public health.

## Materials and methods

### Chemicals

BPA, BPS, and BPF from Sigma-Aldrich (Darmstadt, Germany; S1 Fig) were diluted in dimethyl sulfoxide (DMSO) and added to the adipocyte differentiation medium. They were used at two concentrations (10 nM and 10 μM) for the microarray experiment.

### Adipocyte cell culture

**Fig 1:**
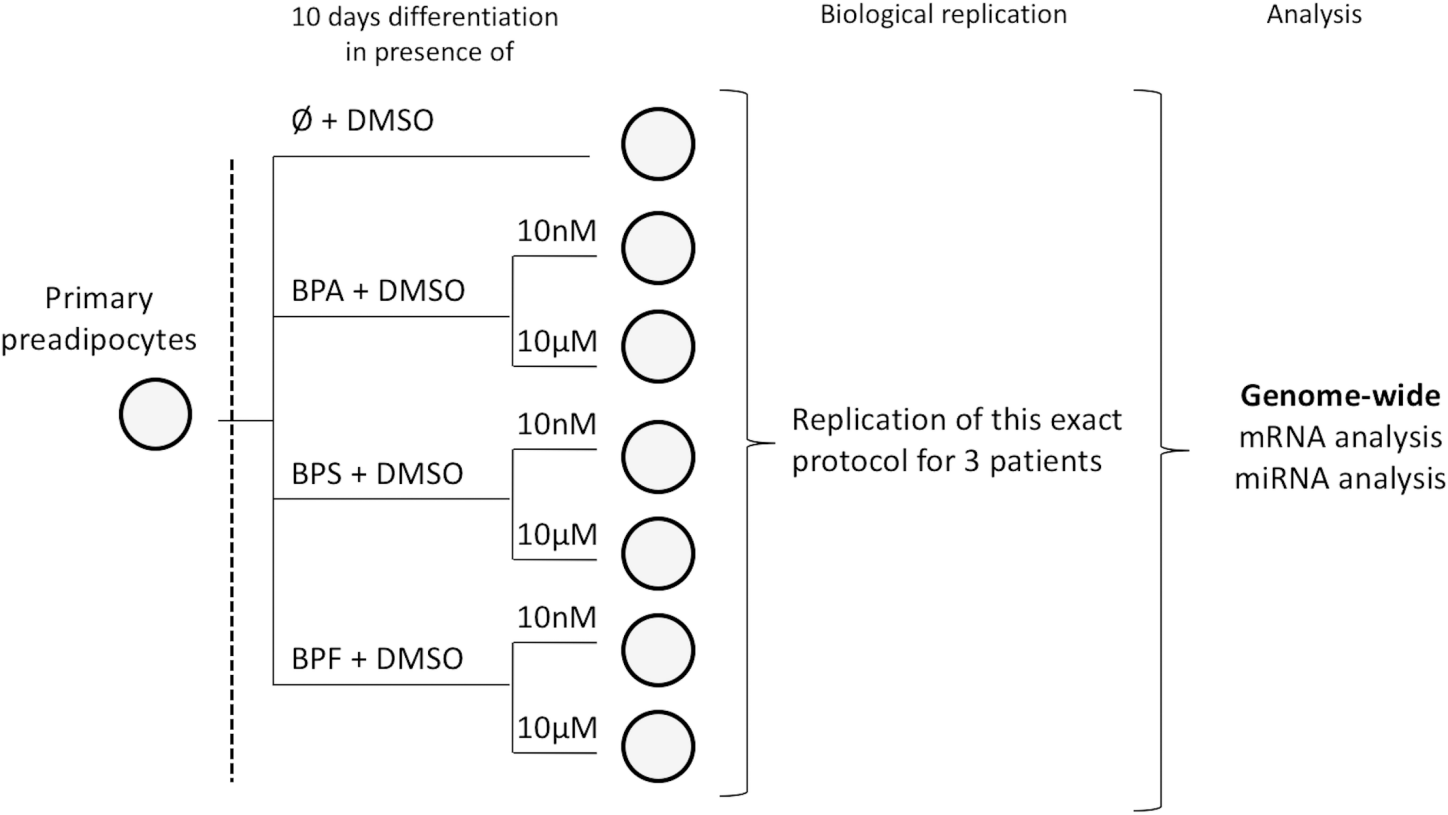
Experimental design and analysis workflow. BPA, BPS, BPF and DMSO: respectively Bisphenol A, S and F and dimethyl sulfoxide.

Primary pre-adipocytes coming from subcutaneous fat of non-diabetic Caucasian female patients were used (Lonza, Basel, Switzerland). Pre-adipocytes are precursor cells that develop into adipocytes when fully differentiated. They were supplied with the differentiation kit and the culture medium. Primary human white subcutaneous pre-adipocytes were cultured in PBM-2 medium. Confluent pre-adipocytes were induced to differentiate with PBM-2 medium supplemented with insulin, dexamethasone, isobutylmethylxanthine and indomethacin (all supplied by Lonza) for ten days according to the instructions of the manufacturer. They were induced to differentiate into adipocytes with either the selected chemical or vehicle (DMSO; Fig 1). After ten days, cells were harvested for total RNA extraction.

### Nucleic acid extraction

Specific kits (Kit Small & Large RNA; Macherey Nagel) were used to extract all species of RNAs. The RNA concentration was determined by absorbance at 260 nm (A260), and the purity was estimated by determining the A260/A230 ratio with a Nanodrop spectrophotometer (Nanodrop Technologies, Wilmington, DE). RNA Integrity Number (RIN) was assessed with a Bioanalyzer (Agilent). This kit allowed the extraction of small and large RNAs with no degradation (RIN>9).

### Microarrays

To measure different species of RNAs, the mRNA and lncRNA microarray SurePrint G3 Human V2 (Agilent Technologies) and miRNA microarray SurePrint G3 miRNA release 19.0 (Agilent Technologies) were used. The microarray experiments from Agilent Technologies were performed and analyzed by the Transcriptomics and Applied Genomics (TAG) team in the Institut Pasteur de Lille (Dr David Hot). This combination of microarrays allowed the detection of mRNAs, but also of long intergenic non-protein coding RNAs, small nucleolar RNAs and miRNAs.

### Data normalization

Henceforth the term probe will be used to refer to a mRNA, lncRNA, snoRNA or miRNA probe. For mRNAs and other long non-coding RNAs, raw data were composed of 62,976 RNA expressions (probes). A first filter consisted in excluding non-expressed probes. To do so, a detection p-value, provided by Agilent which indicated how well the expressions of the gene were detected in a given sample, was used to filter out undetected probes. Probes, which were expressed in a certain proportion of the samples of interest, were kept for further analysis, leading to 28,244 probes (for the study of differentiation) and 22,935 probes (for the study of BPs on differentiation) in the final datasets. In addition, several normalization steps were applied, such as taking into account housekeeping genes or taking the log_2_ of the measures. The normalization was performed with the R package limma [23].

For miRNAs, raw data were composed of a total of 62,344 miRNA probes which represented 2,027 miRNAs (30 probes per miRNA + normalization probes). After a preliminary between-array normalization, the first step of the normalization consisted in estimating a summary measure of the miRNA expression using a linear model to take into account the probe affinity over the 30 probes characterizing each one of the miRNA. Secondly, as for the mRNA data, a detection p-value was used to filter out non-expressed miRNA leading to consider 325 (differentiation) or 483 probes (BPs) as expressed. The normalization was performed with the R package AgiMicroRna [24].

### Statistical analyses

For each one of the product (BPA, BPF, BPS), 12 assays of cell cultures differentiated without any product (negative control, 4 per patient), 12 assays of cell cultures differentiated with 10 nM (2 per patient) or 10 μM of the given product (2 per patient) were compared. For each product, differentially expressed genes were identified with a mixed model, where the variations of a given probe were explained by a fixed effect of the 10 nM concentration and/or the 10 μM concentration in comparison with the control condition, plus the patients which were not of direct interest, were considered as a random effect. P-values were corrected for multiple testing using Benjamini and Hochberg’s method to control the false discovery rate with a 5% threshold. All the analyses were performed with the R package limma.

### Biological analyses

Lists of genes significantly induced or repressed after exposure to BPA were uploaded into Ingenuity Pathway Analysis Software (IPA, Ingenuity Systems, www.ingenuity.com) for biological analysis by comparison with the Ingenuity Knowledge Database. These lists of altered genes were then processed to investigate the functional distribution of the genes, as defined by Gene Ontology. Datasets and known canonical pathway associations were measured by IPA by using a ratio of the number of genes from a dataset that map a specific pathway divided by the total number of genes which map this canonical pathway. Fisher's exact test was used to determine a p-value representing the significance of these associations.

### Taqman RT-PCR

The relative quantification of mRNA was performed with a quantitative RT-PCR assay. 400 ng of total RNA was transcribed into cDNA using the cDNA Archive Kit (Life Technologies, Foster City, CA, USA). Each cDNA sample was analyzed by quantitative real-time PCR (qPCR) using the fluorescent TaqMan 5’-nuclease assays (Life Technologies). The analysis was performed with ViiA 7 detection system and software (Life Technologies). Gene expression was standardized to *POLR2A* expression (2^-ΔCt^).

miRNAs were reverse transcribed using TaqMan Micro RNA Reverse Transcription Kit (Life Technologies). Taqman microRNA assays (Life Technologies) were used for miRNA quantification.

## Results

All microarray data results were deposited on Gene Expression Omnibus website, (http://www.ncbi.nlm.nih.gov/geo/) under the accession number GSE98682 which includes both series of mRNA/lncRNA data (GPL16699) and miRNA data (GPL19730). The samples series providing both the raw and normalized data is accessible through the accession numbers from GSM2609729 to GSM2609851.

### Adipocyte differentiation: mRNA/lncRNA and miRNA expression

Pre-adipocytes coming from subcutaneous fat of three non-diabetic obese females of European origin were used (age: 37, 52 and 38 years old and BMI: 30.3, 39.5 and 32.9 respectively). After ten days of differentiation, as expected, an accumulation of refringent lipid droplets was observed in the cytoplasm of the adipocytes of all three patients, evidencing their ability to fully differentiate.

The heatmap representation of the gene expression in the pre-adipocyte and adipocyte demonstrates a clear-cut set of upregulated and downregulated probes (S2 Fig). Among up-regulated genes classical adipocyte differentiation markers are encountered (LPL, ADIPOQ, FABP4, PLIN4,…) which confirms the successful and complete differentiation of the pre-adipocytes.

For mRNA/lncRNA, 28,244 probes were detected in undifferentiated or differentiated adipocytes. Among these, 122 were significantly differentially up-regulated with a log_2_FC>3 and 275 with a log_2_FC>2; 23 were down-regulated with a log_2_FC<-3 and 114 with a log_2_FC<-2 in differentiated adipocytes compared to undifferentiated adipocytes (S1A Table).

For miRNA, 325 probes were detected in undifferentiated or differentiated adipocytes. Among these, 4 were significantly differentially up-regulated with a log_2_FC>2 and 36 with a log_2_FC>1 and 2 were down-regulated with a log_2_FC<-2 and 23 with a log_2_FC<-1 in differentiated adipocytes compared to undifferentiated adipocytes (S1B Table).

The analysis of probes differentially expressed with a log_2_FC>2 for each subject demonstrated that 56% of differentially expressed probes were shared by at least two patients and 36% by the three patients. (S3A Fig). We also performed principal component analysis (PCA) on all the probes which were detected as expressed. S3 Fig provides the sample representation of the PCA of the mRNA/lncRNA probes and the first two axes allowed us to clearly distinguish the three subjects (S3B Fig) and the differentiation stage (S3C Fig). Nevertheless, this disparity can be turned to our advantage to help us to decipher the shared differentially expressed probes relevant to a general mechanism and not to an individual susceptibility.

For miRNAs, the up or down-regulation during adipocyte differentiation observed was less obvious with lower fold changes and a very high variability between patients. With a log_2_FC>2, no miRNA probe was shared between the three patients (S4A Fig). Shared miRNA probes were detected only with a log_2_FC>1 (S4B Fig). Only 7% of probes unregulated with a log_2_FC>1 in the first patient were also up-regulated with a log_2_FC>1 in the other two patients and 42% of probes up-regulated with a log_2_FC>1 were down-regulated in at least one of the other two patients. In opposition to the PCA of the mRNA/lncRNA probes, the three patients and the differentiation stage could not be clearly distinguished using the first two principal components for the miRNA probes (S4C and S4D Fig).

On the mRNA/lncRNA microarray, the expression of a specific gene can be measured with several probes. The probes refereeing to the same gene were not aggregated, but analyzed separately since they might capture alternative splicing. Among the 20 most differentially expressed mRNA probes in differentiated adipocytes according to p-values, only up-regulated genes were found (S1A Table). Classical genes of adipocyte differentiation, such as *LPL*, *PLIN1/4*, *ADIPOQ* or *FABP4* were found within this top list. For miRNA probes, the observed fold changes were lower along with less significant p-values. In contrast to mRNAs, both up and down-regulated miRNAs were found (S1B Table). Two long intergenic non-coding RNAs (LINC01140, LINC01088) were up-regulated in differentiated adipocytes and one (LINC01048) was down-regulated compared to pre-adipocytes (S1A Table).

### Alteration of the global profiles by exposure to bisphenols

#### mRNA and lncRNA

After an exposition to 10 nM of BPA (or BPS or BPF) during adipocyte differentiation, using a mixed model, 846 probes coding for mRNA and lncRNA (respectively 1,173 and 216) were found to be significantly down-regulated and 417 (respectively 367 and 621) up-regulated when compared to the control cells without bisphenol (S2 Table). After the exposition to the higher dose of 10 μM of BPA (or BPS or BPF) during adipocyte differentiation, using a mixed model, 774 probes (respectively 748 and 1,228) were found to be significantly down-regulated and 1106 (respectively 323 and 989) up-regulated when compared to the control cells without bisphenol (S2 Table). Among the probes detected using mRNA/lncRNA arrays, some long intergenic non-coding RNAs and some small nucleolar RNAs were differentially expressed after exposition to the bisphenols during differentiation (S2 Table).

Considering all the deregulated probes, several of these deregulated probes were shared between concentrations (Table 1) and between bisphenols with the majority in the same direction (Table 2). For example, A_33_P3321657 tagging *HSPG2* is in the top 20 of most differentially expressed probes and down-regulated for BPS and BPF at both concentrations.

**Table 1:** Numbers of differentially down and up-regulated (mRNA/lncRNA) probes amongst the 22,935 probes after the exposition of the pre-adipocytes to 10 nM or 10 μM of bisphenol shared between the concentrations for each bisphenol.

		10 nM	shared	10 μM
**BPA**	**up**	846	211	774
	**down**	417	126	1,106
**BPS**	**up**	1,173	387	748
	**down**	367	60	323
**BPF**	**up**	216	46	1,228
	**down**	621	133	989

**Table 2:** Number of differentially down and up-regulated (mRNA/lncRNA) probes amongst the 22,935 probes after the exposition of the pre-adipocytes to 10 nM or 10 μM of bisphenol (A, F and S) during differentiation shared between BPA and BPS or BPF.

		**BPS 10 nM**		**BPF 10 nM**	
		1,173 down	367 up	216 down	621 up
**BPA 10nM**	846 down	524	0	43	4
	417 up	0	65	0	77
		**BPS 10 μM**		**BPF 10 μM**	
		748 down	323 up	1,228 down	989 up
**BPA 10μM**	774 down	260	2	226	1
	1,106 up	3	110	8	223

**Fig 2:**
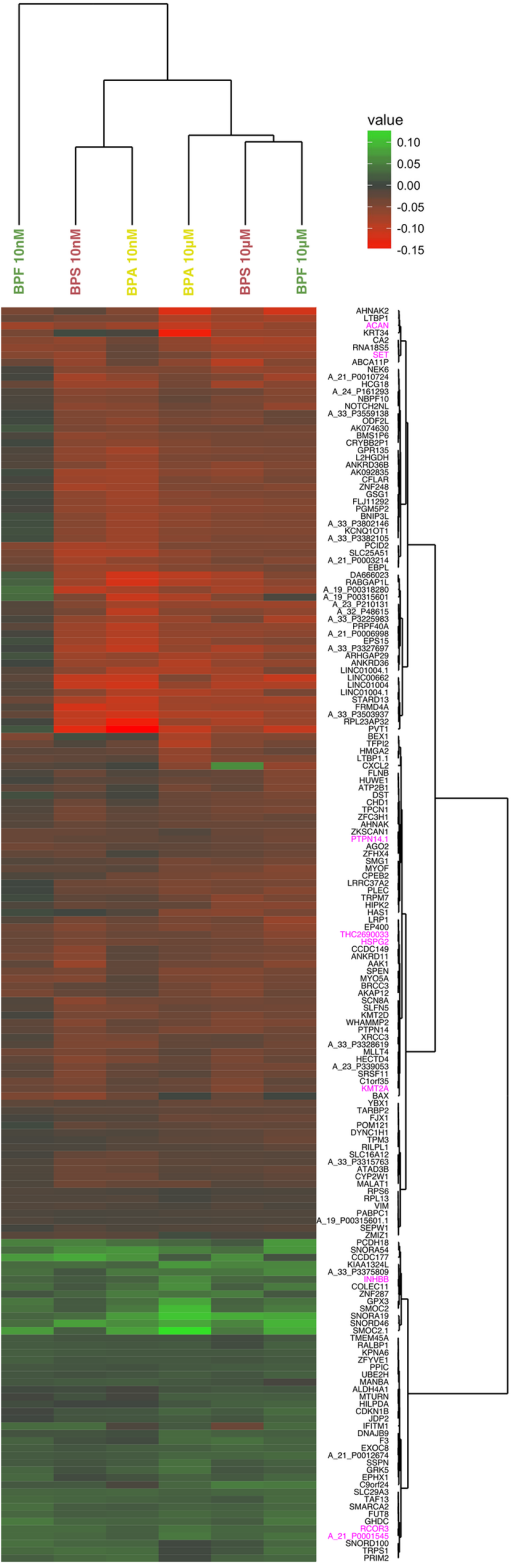
Heatmap representation of significantly differentially expressed genes in the three bisphenols at 10 nM and 10 μM. The represented values are log_2_ fold changes of the ratio of the expression in a given bisphenol and concentration over the control condition which is differentiated without any bisphenol. Genes (probes) highlighted in pink are significant for all concentrations of all bisphenols.

To complement the previous results, we represented the global expression profiles of the significantly differentially expressed genes according to the three bisphenols in a heatmap (Fig 2). What is sticking is the great homogeneity within the profiles. Indeed when a gene is over or underexpressed in one concentration of a bisphenol, it tends to have a similar response to the others, both in terms of direction and magnitude of effect. It is worth noting that BPF at 10 nM seems to behave slightly differently and shows an overall reduced magnitude of effect, which might suggest that a higher dose of BPF is required to have a similar effect as BPA and S in terms of expression profile.

#### miRNA

After an exposition to 10 nM of BPA (or BPS or BPF) during adipocyte differentiation, 39 probes coding for miRNA (respectively 61 and 0) were found to be significantly down-regulated and 33 (respectively 40 and 12) up-regulated when compared to the control cells without bisphenol (S2 Table). After the exposition to the higher dose of 10 μM of BPA (or BPS or BPF) during adipocyte differentiation, 18 probes (respectively 11 and 50) were found to be significantly down-regulated and 13 (respectively 8 and 48) up-regulated when compared to the control cells without bisphenol (S2 Table).

miRNA probes shared between concentrations and between bisphenols are displayed in Table 3 and Table 4. As for mRNA, shared probes are dysregulated in the same direction. The most significant fold-changes values for miRNA are higher than fold changes observed for mRNA/lncRNA.

**Table 3:** Numbers of differentially down and up-regulated miRNA probes amongst the 483 probes after the exposition of the pre-adipocytes to 10 nM or 10 μM of bisphenol shared between the concentrations for each bisphenol.

		10 nM	shared	10 μM
**BPA**	**up**	39	2	18
	**down**	33	0	13
**BPS**	**up**	61	3	11
	**down**	40	3	8
**BPF**	**up**	0	0	50
	**down**	12	4	48

**Table 4:** Number of differentially down and up-regulated miRNA probes amongst the 483 probes after the exposition of the pre-adipocytes to 10 nM or 10 μM of bisphenol (A, F and S) during differentiation shared between BPA and BPS or BPF.

		**BPS 10 nM**		**BPF 10 nM**	
		61 down	40 up	0 down	12 up
**BPA 10nM**	39 down	32	0	0	0
	33 up	0	26	0	0
		**BPS 10 μM**		**BPF 10 μM**	
		11 down	8 up	50 down	48 up
**BPA 10μM**	18 down	7	0	3	0
	13 up	0	5	0	2

**Fig 3:**
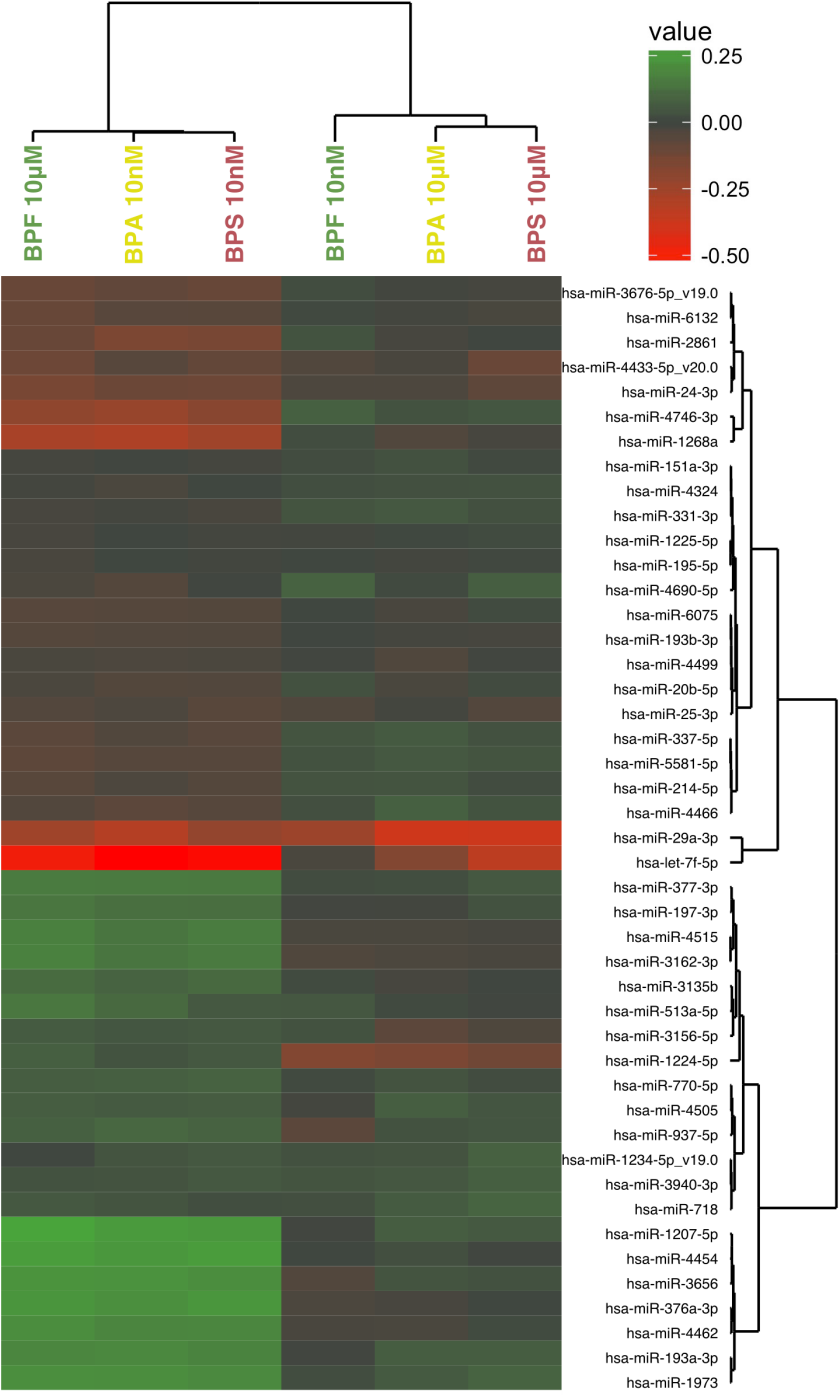
Heatmap representation of significantly differentially miRNA in the three bisphenols at 10 nM and 10 μM. The represented values are log_2_ fold changes of the ratio of the expression in a given bisphenol and concentration over the control condition which is differentiated without any bisphenol.

In addition, we represented the global expression profiles of the significantly differentially expressed miRNAs according to the three bisphenols in a heatmap (Fig 3). We observed, just like for mRNAs/lncRNAs, an overall homogeneity within the expression profiles, although we clearly see a separation in the clustering. As previously stated for the mRNAs/lncRNAs, BPF at 10 nM shows an overall reduced expression and the response to BPF at 10 μM is extremely similar to the response to BPA and S at 10nM.

#### Analysis of biological functions

Similarities between the alterations of the profile were assessed using Ingenuity Pathway Analysis software. The mRNA differentially expressed in response to 10 nM and 10 μM exposure of all three bisphenols (BPs) were analyzed: top canonical associated pathways, upstream regulators as well as related diseases and disorders are displayed in Table 5. We found shared enriched pathways for all three BPs. For instance, eIF2 signaling was among the top canonical pathways both for BPA and BPF; ESR1, MYCN or MYC were found as upstream regulators; and “cancer” as well as “organismal injury and abnormalities” were shared between the three products. The same analysis performed on the lists of differentially expressed mRNA probes of all three bisphenols (BPs) for the two concentrations gave similar results, suggesting general dose-independent mechanisms.

**Table 5:** Enrichment analysis using Ingenuity Pathway Analysis on the lists of mRNA probes differentially expressed after exposition to the three bisphenols A, F and S at 10 nM and 10 μM.

	**10nM BPA**		**10nM BPS**			**10nM BPF**	
	Name	p-value	Name		p-value	Name	p-value
Top canonical pathway	EIF2 Signaling	1.40E-03	Mouse Embryonic Stem Cell Pluripotency		8.50E-05	EIF2 Signaling	1.05E-03
	NAD Biosynthesis III	1.54E-02	Leptin Signaling in Obesity		1.05E-04	Acute Phase Response Signaling	1.31E-03
	Aspartate Degradation II	2.11E-02	Role of NFAT in Cardiac Hypertrophy		1.65E-04	Inhibition of Matrix Metalloproteases	1.53E-03
	Sorbitol Degradation I	3.35E-02	Human Embryonic Stem Cell Pluripotency		3.42E-04	Interferon Signaling	5.91E-03
	Netrin Signaling	4.08E-02	PPAR/RXR Activation		1.05E-03	Heme Degradation	6.09E-03
Top Upstream Regulators	MYCN	1.19E-04	HNF4A		2.76E-06	TP53	1.14E-07
	miR-124-3p (and miRNAs w/seed AAGGCAC)	9.46E-04	ESR1		1.86E-05	MYC	1.31E-06
	FAAH	1.71E-03	OSM		3.21E-05	HRAS	2.04E-06
	TP53	1.93E-03	P4HB		7.74E-05	TGFB1	3.63E-06
	phosphate	2.42E-03	MYCN		1.40E-04	ESR1	4.69E-06
Diseases and Disorders	Cancer	3.35E-02–3.56E-12	Cancer		1.14E-02–7.03E-14	Cancer	5.75E-03–4.62E-09
	Organismal Injury and Abnormalities	3.35E-02–3.56E-12	Gastrointestinal Disease		1.14E-02–7.03E-14	Organismal Injury and Abnormalities	6.08E-03–4.62E-09
	Gastrointestinal Disease	3.35E-02–8.11E-09	Hepatic System Disease		1.10E-02–7.03E-14	Infectious Diseases	6.04E-03–1.56E-08
	Hepatic System Disease	1.80E-02–8.11E-09	Organismal Injury and Abnormalities		1.14E-02–7.03E-14	Gastrointestinal Disease	6.06E-03–4.27E-08
	Reproductive System Disease	3.35E-02–1.41E-07	Renal and Urological Disease		1.04E-03–3.70E-09	Reproductive System Disease	4.51E-03–1.09E-05
	**10μM BPA**		**10μM BPS**			**10μM BPF**	
	Name	p-value	Name	p-value		Name	p-value
Top canonical pathway	Axonal Guidance Signaling	8.60E-06	Molecular Mechanisms of Cancer	1.09E-03		Death Receptor Signaling	3.16E-04
	Remodeling of Epithelial Adherens Junctions	1.07E-04	Aryl Hydrocarbon Receptor Signaling	3.66E-03		phagosome maturation	1.08E-03
	Molecular Mechanisms of Cancer	2.66E-04	Glucocorticoid Receptor Signaling	3.72E-03		Mitochondrial Dysfunction	1.49E-03
	Sertoli Cell-Sertoli Cell Junction Signaling	5.92E-04	Hepatic Fibrosis / Hepatic Stellate Cell Activation	4.97E-03		tRNA Charging	1.63E-03
	Acute Phase Response Signaling	6.69E-04	Cell Cycle: G2/M DNA Damage Checkpoint Regulation	6.85E-03		Oxidative Phosphorylation	2.27E-03
Top Upstream Regulators	beta-estradiol	6.69E-13	ESR1	1.14E-10		TP53	2.10E-09
	Vegf	8.05E-13	PDGF BB	3.41E-10		IND S1	5.50E-08
	TGFB1	9.82E-13	TP53	7.02E-10		IND S7	1.17E-07
	dexamethasone	1.14E-11	tretinoin	1.47E-09		CD 437	1.61E-07
	TNF	1.38E-11	dexamethasone	3.13E-09		MEL S3	1.86E-07
Diseases and Disorders	Cancer	8.16E-04–8.06E-22	Cancer	1.01E-03–7.01E-19		Cancer	6.27E-03–1.40E-15
	Organismal Injury and Abnormalities	8.16E-04–8.06E-22	Organismal Injury and Abnormalities	1.01E-03–7.01E-19		Organismal Injury and Abnormalities	6.27E-03–1.40E-15
	Gastrointestinal Disease	7.98E-04–1.15E-16	Gastrointestinal Disease	9.91E-04–9.85E-17		Gastrointestinal Disease	6.27E-03–5.35E-13
	Reproductive System Disease	7.25E-04–2.29E-16	Reproductive System Disease	7.69E-04–9.39E-15		Reproductive System Disease	6.00E-03–7.57E-12
	Endocrine System Disorders	6.47E-04–1.67E-10	Hepatic System Disease	2.97E-04–2.42E-14		Infectious Diseases	5.45E-03–5.58E-09

For each bisphenol, the five most significantly enriched canonical pathways, top upstream regulators as well as diseases and disorders are displayed with the corresponding p-values. The enrichment is based on the comparison between the number of dysregulated gene involved in a given function in the dataset compared to the number expected by chance. The displayed range of p-values corresponds to the range of p-values encountered for the different annotations in this category.

#### Probes shared by the three bisphenols

The selection of the probes differentially expressed at a concentration of 10 nM for all three BPs led to highlight 40 (37) mRNA probes (genes), but no miRNA. Among these 40 probes, 16 were up-regulated and 24 were down-regulated (Table 6). Cytoskeleton and ribosomal proteins were found within this set of genes suggesting that crucial mechanisms of cell regulation and of protein traduction could be disrupted in presence of low dose bisphenols. The selection of the probes differentially expressed at a concentration of 10 μM for all three BPs led to highlight 161 (134) mRNA probes (genes), and only one miRNA (hsa-miR-4655-3p). Among these 161 probes, 33 were up-regulated and 128 were down-regulated (S3 Table). If only the probes differentially expressed in both concentrations of all three BPs were selected, we highlighted 12 (10) mRNA probes (genes). Among these ten genes, two were up-regulated and eight were down-regulated; they are highlighted with a grey background in Table 6 (10 nM) and with yellow filling in S3 Table (10 μM). From our analyses, only one miRNA (hsa mir-4655-3p) was differentially expressed in all three BPs, but only at a high concentration of 10 μM for BPA and at both concentrations for BPS and BPF.

**Table 6:** List of differentially expressed probes deregulated for the three bisphenols A, F and S at 10 nM.

			BPA		BPS		BPF	
ProbeName	GeneName	Entrez Gene Name	log_2_FC	P-value	log_2_FC	P-value	log_2_FC	P-value
A_23_P44139	PRIM2	primase (DNA) subunit 2	**0.216**	4.81E-05	**0.189**	3.78E-04	**0.131**	8.26E-03
A_33_P3280044	ANKRD11	ankyrin repeat domain 11	-0.227	7.52E-05	-0.326	5.10E-07	-0.264	7.51E-04
A_19_P00315601	XLOC_014512		-0.687	8.34E-05	-0.292	6.91E-04	0.224	1.39E-02
A_24_P232790	CCDC177	coiled-coil domain containing 177	**0.445**	2.81E-04	**0.567**	3.16E-04	**0.482**	1.33E-03
A_23_P307310	ACAN	aggrecan	-0.488	3.01E-04	-0.4	6.10E-04	-0.508	7.26E-04
A_33_P3321657	HSPG2	heparan sulfate proteoglycan 2	-0.367	4.39E-04	-0.441	3.23E-08	-0.293	7.05E-05
A_23_P90143	RPL13A	ribosomal protein L13a	-0.165	4.52E-04	-0.156	9.83E-04	-0.133	5.30E-03
A_33_P3278407	AFDN	afadin, adherens junction formation factor	-0.189	5.40E-04	-0.249	3.09E-05	-0.196	1.40E-03
A_21_P0014132	THC2690033		-0.177	5.68E-04	-0.241	1.60E-05	-0.171	5.33E-04
A_24_P148235	RPS27	ribosomal protein S27	-0.134	7.61E-04	-0.115	4.68E-03	-0.137	4.14E-03
A_21_P0013977	PCID2	PCI domain containing 2	-0.395	8.31E-04	-0.429	1.07E-03	-0.273	9.76E-03
A_23_P208706	BAX	BCL2 associated X, apoptosis regulator	-0.257	8.38E-04	-0.467	5.43E-04	-0.359	5.66E-03
A_23_P258698	MANBA	mannosidase beta	**0.149**	9.52E-04	**0.135**	5.83E-04	**0.111**	1.03E-02
A_19_P00323413	PTPN14	protein tyrosine phosphatase, non-receptor type 14	-0.159	9.97E-04	-0.156	3.87E-03	-0.192	2.26E-03
A_23_P153964	INHBB	inhibin beta B subunit	**0.196**	1.48E-03	**0.136**	1.25E-02	**0.206**	3.90E-03
A_23_P376599	RALBP1	ralA binding protein 1	**0.127**	1.65E-03	**0.143**	9.60E-04	**0.155**	1.01E-03
A_24_P142228	RPL13	ribosomal protein L13	-0.161	2.07E-03	-0.14	5.52E-03	-0.137	4.01E-03
A_23_P69521	CCNI	cyclin I	-0.127	2.14E-03	-0.132	9.97E-04	-0.113	9.58E-03
A_33_P3361746	TRPS1	transcriptional repressor GATA binding 1	**0.182**	2.18E-03	**0.195**	3.70E-03	**0.168**	1.19E-02
A_23_P128956	ZFYVE1	zinc finger FYVE-type containing 1	**0.11**	3.24E-03	**0.099**	1.72E-02	**0.152**	2.38E-03
A_23_P214666	RPS18	ribosomal protein S18	-0.174	3.35E-03	-0.144	1.74E-02	-0.145	1.66E-02
A_33_P3390570	KMT2A	lysine methyltransferase 2A	-0.155	4.17E-03	**-**0.253	2.03E-05	-0.199	5.33E-04
A_33_P3344831	TMEM45A	transmembrane protein 45A	**0.128**	5.00E-03	**0.128**	8.13E-03	**0.183**	4.77E-04
A_23_P35148	TAF13	TATA-box binding protein associated factor 13	**0.179**	5.16E-03	**0.2**	6.81E-03	**0.225**	9.50E-04
A_23_P217015	SET	SET nuclear proto-oncogene	-0.175	5.19E-03	-0.439	7.38E-09	-0.35	2.28E-03
A_23_P77103	SORD	sorbitol dehydrogenase	-0.184	5.22E-03	-0.174	7.63E-04	-0.18	2.69E-03
A_21_P0001545	LOC149351		**0.13**	6.60E-03	**0.181**	2.69E-03	**0.201**	2.13E-03
A_23_P207387	GHDC	GH3 domain containing	**0.139**	7.08E-03	**0.151**	1.13E-02	**0.241**	7.23E-04
A_19_P00317052	RNF213	ring finger protein 213	-0.183	7.40E-03	-0.164	1.59E-02	-0.164	1.89E-02
A_23_P46871	SLC29A3	solute carrier family 29 member 3	**0.158**	7.57E-03	**0.149**	1.17E-02	**0.157**	1.40E-02
A_21_P0000240	SNORD100	small nucleolar RNA, C/D box 100	**0.138**	8.18E-03	**0.142**	1.61E-02	**0.189**	1.14E-02
A_23_P138435	ZMIZ1	zinc finger MIZ-type containing 1	-0.126	1.04E-02	-0.153	1.65E-03	-0.125	1.66E-02
A_33_P3234667	ZKSCAN1	zinc finger with KRAB and SCAN domains 1	-0.152	1.07E-02	-0.184	1.08E-03	-0.185	7.75E-03
A_24_P276816	RCOR3	REST corepressor 3	**0.119**	1.07E-02	**0.174**	1.42E-03	**0.174**	2.27E-03
A_33_P3225760	PCDH18	protocadherin 18	**0.2**	1.08E-02	**0.285**	4.89E-04	**0.278**	5.24E-04
A_33_P3210278	SYNE2	spectrin repeat containing nuclear envelope protein 2	-0.196	1.17E-02	-0.175	1.16E-02	-0.166	1.14E-02
A_23_P123563	RPS6	ribosomal protein S6	-0.101	1.18E-02	-0.131	2.09E-03	-0.143	1.80E-03
A_23_P501822	JUP	junction plakoglobin	-0.154	1.31E-02	-0.233	4.65E-03	-0.26	1.36E-03
A_24_P128563	KPNA6	karyopherin subunit alpha 6	**0.105**	1.64E-02	**0.118**	1.14E-02	**0.137**	3.65E-03
A_23_P161190	VIM	vimentin	-0.131	1.68E-02	-0.179	7.25E-03	-0.151	6.93E-03

Fold changes (FC) are actually log_2_ transformed fold changes, therefore if a FC is positive (in bold) the probe is over-expressed in the tested condition compared to the control, whereas it is under-expressed when a FC is negative. Probes differentially expressed for both concentrations (10 nM and 10 μM) are displayed with a grey background.

In addition, we searched with IPA for potential upstream regulators of the latter set of ten genes differentiated for the three bisphenols at both concentrations (Table 7). Among the upstream regulators, beta-estradiol and ESR1 already known to be targeted by BPA were found as potential targets for all three BPs.

**Table 7:** Upstream regulators provided by Ingenuity Pathway Analysis in the 10 genes differentially expressed at both concentrations (10 nM and 10 μM) for the three bisphenols (A, F and S).

Upstream Regulator	Molecule Type	P-value of overlap	Target molecules in dataset
beta-estradiol	chemical—endogenous mammalian	4.82E-03	CCNI,INHBB,SET,SORD
APP	other	4.98E-03	HSPG2,KMT2A,SET
ESR1	ligand-dependent nuclear receptor	1.51E-02	CCNI,INHBB,SORD
Vegf	group	1.02E-03	ACAN,INHBB,KMT2A
TGFB1	growth factor	2.87E-02	ACAN,HSPG2,INHBB
PAX3	transcription regulator	3.10E-03	HSPG2,PTPN14
AGT	growth factor	1.45E-02	HSPG2,INHBB
GDF9	growth factor	1.11E-04	HSPG2,INHBB
dihydrotestosterone	chemical—endogenous mammalian	2.09E-02	CCNI,INHBB
LY294002	chemical—kinase inhibitor	1.83E-02	ACAN,INHBB
IL1B	cytokine	4.99E-02	ACAN,HSPG2

In order to validate the microarray data by quantitative PCR, we first screened classical housekeeping gene expressions. *POLR2A* (Polymerase (RNA) II Subunit A) demonstrated no significant differential expression for the three bisphenols at both doses and was chosen as housekeeping gene (S4 Table). Taqman qPCR analysis was performed using “best coverage” probes recommended by the manufacturer (S5 Table).

The direction and values of the expression fold-changes obtained by qPCR were consistent with the data from the microarray analysis for eight of the ten genes studied (S6 Table). *SET* and *SORD* showed an up-regulation when analyzed by Taqman qPCR which is opposite to the microarray data. In the microarray analysis, only one probe among three annotated for *SET* and *SORD* was differentially expressed therefore different transcripts were evaluated by microarray probes or by qPCR probes: this explains the observed discrepancies. Without *SET* and *SORD* probes, the correlation coefficient obtained by Spearman analysis was r = 0.708 (p = 2.56 x 10^−8^) in expression between the data from the microarray analysis and from Taqman qPCR (S6 Table). mir-4655-3p expression analyzed by Taqman was very low and was only detectable above the threshold of 35 cycles.

We integrated data from the Comparative ToxicoGenomics Database (CTD, http://ctdbase.org/) which tells us if BPA has already been shown to modulate the expression of a given gene. CTD provides manually curated information about chemical–gene/protein interactions, coming from published microarray data. BPA is highly represented but no association has been curated for BPS and BPF yet. Among the 40 probes deregulated for the three bisphenols at 10 nM, all the probes, with the exception of the probes *SNORD100* and *ANKRD11*, were found to interact with BPA in this database.

## Discussion

We have intended here to make progress in toxicology analyses of common chemicals present in the human environment, by the use of human primary cells chronically exposed to these chemicals, at a concentration similar to the range found in human fluids in many studies [18], with a hypothesis-free genomic approach analyzing both coding and non-coding RNA levels. We considered this unbiased strategy important to analyze the differential effects of BPA and of its substitutes, BPS and BPF, on human cellular genomic profiles.

We first validated the use of primary cell cultures derived from three different patients. Indeed, mRNA/lncRNA expression induced by pre-adipocyte differentiation was very consistent between subjects. Despite some expected variability, we were indeed able to highlight classical markers of differentiation (*LPL*, *FABP4*, *ADIPOQ*, *PLIN1/4* …) demonstrating both the achievement of differentiation of the cells and the validity of our design. Concerning the miRNA study, we showed that the miRNA profile for adipocyte differentiation is very heterogeneous between individuals. Nevertheless, among the top probes (according to p-values) differentially expressed during pre-adipocyte differentiation, some are consistent with existing literature. miR-26a, which was down-regulated in our study, induces characteristics of brown adipocytes during human adipocyte differentiation [25]. miR-30c up-regulated in our study, was shown to promote human adipocyte differentiation [26]. miR-136, down-regulated in our study, was up-regulated during chondrogenesis of human adipose-derived stem cells suggesting that it can be considered as a player in the switch from white adipocyte to chondrocyte [27].

More importantly, these three cell cultures were sufficient to detect significantly differentially expressed RNA species in response to bisphenols, demonstrating an effect of bisphenols even at “low doses” on cellular metabolism. We showed here that both BPA and its substitutes BPS and BPF, induced a dysregulation of major regulators in cell homoeostasis, which is illustrated by highlighting cancer and translation pathways, whatever the concentration used. Among the mRNA/lncRNA differentially expressed probes for BPA, 46% were shared with BPS and only 10% with BPF. For miRNA probes, 80% were shared between BPA and BPS while none were shared between BPA and BPF. Interestingly, among the mRNA/lncRNA differentially expressed probes for BPA at 10 nM, 46% were shared with probes differentially expressed for BPF, when BPF was used at 10 μM. These results suggest that BPS effect is closer than BPF to BPA effect and that a higher concentration of BPF could be required for an effect on the same probes. Nevertheless, when focusing on the probes deregulated for the three bisphenols, we highlighted extracellular matrix and cytoskeleton genes, transcription regulators, cyclins: all genes implied in cell metabolism regulation. In addition, the analysis of upstream regulators of the set of ten genes differentiated for the three bisphenols at both concentrations highlighted hormones or hormone-like chemicals (beta-estradiol, dihydrosterone) as well as ESR1 (estrogen receptor 1). This suggests that BPS and BPF, can be suspected to interfere, just like BPA, with hormonal regulation, and therefore deserve to undergo the same restrictions from a regulation standpoint.

In this regard, we highlighted an impairment of basic pathways of cell metabolism by bisphenols, such as eIF2 (Eukaryotic Initiation Factor 2) signaling; eIF2 is a eukaryotic initiation factor required in the initiation of translation. Moreover, among the more represented disease categories, corresponding to differentially expressed species, we found “cancer” and “organismal injuries and abnormalities”. It is worth noting that these oncogenic features cannot be attributed to the cellular model since we have used primary cells. Surprisingly, in our conditions, neither adipogenesis nor inflammation probes were overrepresented as it was observed in other cellular adipocyte models [28] [29]. BPA was demonstrated to significantly enhanced adipogenesis via an ER-mediated pathway [29]. BPA has been first considered a weak estrogen, based on its low binding affinity to the nuclear estrogen receptors, however there is also evidence of a potent BPA-estrogenic action through non-classical estrogen pathways e.g. *via* ERalpha located outside the nucleus [30]. In beta cells 1 nM BPA regulates insulin gene transcription via ERalpha in an estrogen response element independent manner [31]. Extranuclearly located ERbeta mediates rapid actions of BPA (1 nM) as well [32]. The analysis of upstream regulators of the set of ten genes differentiated for the three bisphenols at both concentrations highlighted ESR1 (estrogen receptor 1 or ERalpha). Therefore, our results suggest that BPA, and its substitutes BPS and BPF, are potent estrogens acting at nanomolar concentrations via ERalpha. Further studies are required to determine whether this could occur through the activation of ER-extranuclear signaling pathways, as demonstrated in other models [33].

The originality of our study resides in the origin of our cells: widely used 3T3-L1 [28] [34] are murine pre-adipocytes, in addition many studies have demonstrated an effect only in a micromolar range [35] [36]. More importantly, the differentiation stage is very crucial: in a recent study from Boucher *et al*. [37], it was nicely demonstrated that 10 nM BPS induces a significant increase of 1.6-fold in *PPAR-gamma* expression at day 4 in primary human pre-adipocytes, but this over-expression was no longer detectable at day 10. In addition, in this study from Boucher *et al*., other adipogenic markers such as *FABP4*, *LPL*, *PLIN* were significantly increased only by the use of a 25 μM concentration of BPS.

It is worth mentioning that some long intergenic non-coding RNAs and some small nucleolar RNAs were differentially expressed after the exposition to the bisphenols during differentiation. To our knowledge, there is few data reporting the induction of long intergenic non-coding RNAs or small nucleolar RNAs by any bisphenol. The long non-coding RNA HOTAIR was induced in breast cancer cells by BPA [38]. Fifteen small nucleolar RNAs with C/D motif (SNORD) were shown to be reduced in cultures of primary prostate epithelial cells treated with BPA [39]. SNORD are noncoding, small nucleolar RNAs known to regulate ribosomal RNA assembly and function, suggesting that bisphenol A and its substitutes may also act through epigenetic modifications, as already reported [40].

Interestingly, two references were frequently found along comparison of our data with the Comparative ToxicoGenomics Database. The first one is from Ali *et al*. [41] and describes a microarray experiment on a rat seminiferous tubule culture model exposed for several days with low dose bisphenol A (1 nM and 10 nM) and intersects 31 of our 40 probes. The culture conditions are very similar to ours and it is striking that in a different model undergoing DNA reprogramming such as meiosis, the same genes can be affected by BPA. The second reference was found for 8 of our 40 probes, and describes the ovarian transcriptome of two fish species exposed to BPA [42].

Among the genes identified by this study as bisphenol targets, *inhibin beta B subunit* (*INHBB*) has drawn our attention because it has already been described as a target of estrogens [43]. The beta B subunit forms a homodimer activin B, and in combination with the beta A subunit forms a heterodimer activin AB, both of which stimulate FSH secretion [44]. INHBB produced in adipocytes may play a role in the metabolic syndrome, since it was demonstrated that its expression is high in human adipocytes, reduced by weight loss, and correlates with factors implicated in metabolic disease [45]. In addition, INHBB was shown to inhibit lipolysis in adipocytes [46] and decrease thermogenesis in primary cultures of mouse white adipocytes [47]. Considering that INHBB may be used as a marker of spermatogenesis function and male infertility [48], our results strengthen the need of regulations that guarantee a high level of protection of human health.

In conclusion, our results suggest that human primary adipocytes undergoing chronic exposure to BPA or its substitutes BPS and BPF, during differentiation, present deleterious effects on their transcriptome even at a “low” dose (e.g. frequently encountered in a general population). The upstream regulators of the deregulated genes belong to hormonal chemicals and the most highlighted pathways are related to oncogenesis. We noticed an interaction with different families of non-coding RNAs (miRNA, long non-coding and small nucleolar RNAs) suggesting an effect of the three bisphenols on the regulation of gene transcription and also with various aspects of post-transcriptional mRNA processing. Altogether these data suggest that more caution should be taken for the use of BPA and its so-called “substitutes” as previously unsuspected impaired cellular event could be initiated even at a low dose.

## Supporting information

S1 Fig Chemical characteristics of bisphenol A and its two analogs F and S. (TIF)

S2 Fig Heatmap representation of significantly differentially expressed genes between the pre-adipocyte and the differentiated adipocyte. The difference between the average expression in the pre-adipocyte and the adipocyte with the global expression is represented.
(TIF)

S3 Fig Variability between patients (mRNA/lncRNA). A) Venn diagram of differentially expressed probes for each patient before and after ten days of differentiation, a probe is considered as differentially expressed if log_2_FC>2. B) Individual representation of the first two axes of the principal component analysis on all probes for each patient before and after ten days of differentiation (colored according to patient). C) Individual representation of the first two axes of the principal component analysis on all probes for each patient before and after ten days of differentiation (colored according to differentiation stage).
(TIF)

S4 Fig Variability between patients (miRNA). A) Venn diagram of differentially expressed probes for each patient before and after ten days of differentiation, a probe is considered as differentially expressed if log_2_FC>2. B) Venn diagram of differentially expressed probes for each patient before and after ten days of differentiation, a probe is considered as differentially expressed if log_2_FC>1. C) Individual representation of the first two axes of the principal component analysis on all probes for each patient before and after ten days of differentiation (colored according to patient). D) Individual representation of the first two axes of the principal component analysis on all probes for each patient before and after ten days of differentiation (colored according to differentiation stage).
(TIF)

S1 Table Probes with up-regulated or down-regulated expression for mRNA/lncRNA (Spreadsheet A) or miRNA (Spreadsheet B) in differentiated adipocytes. Fold changes are log_2_ transformed fold changes (log_2_FC) of the expression in differentiated adipocytes compared to undifferentiated pre-adipocytes (positive when the probe is over-expressed; negative when the probe is under-expressed). P-values are corrected for multiple testing using Benjamini and Hochberg’s method (false discovery rate<5%). Only probes with log_2_FC>2 or log_2_FC<-2 for mRNA (A) and log_2_FC>1 or log_2_FC<-1 for miRNA (B) are listed. Gene names (A) or miRNA symbols (B) have been determined with Ingenuity Pathway. D indicates the presence of a duplication: two different probes are tagging the same gene. Long non-coding RNA data are displayed with a blue filing. ID corresponds to the probe id.
(XLSX)

S2 Table List of RNA (mRNA and lncRNA) and miRNA probes differentially expressed for the three bisphenols (A, F and S). Gene names (A) or miRNA symbols (B) have been determined with IPA. D indicates the presence of a duplication: two different probes are tagging the same gene. Fold changes (FC) are actually log_2_ transformed fold changes (log_2_FC), therefore if a FC is positive the probe is over-expressed in the tested condition compared to the control, whereas it is under-expressed when a FC is negative. In addition, when a FC is equal to +1 or -1, the probe is twice as over or under-expressed. P-values are corrected for multiple testing using Benjamini and Hochberg’s method to control the false discovery rate with a 5% threshold. Long intergenic non-coding RNAs data are displayed with a blue filing and small nucleolar RNAs data are displayed with a pink filing. ID corresponds to the probe id.
(XLSX)

S3 Table List of differentially expressed probes deregulated for the three bisphenols A, F and S at 10 μM. Fold changes (FC) are actually log_2_ transformed fold changes, therefore if a FC is the probe is over-expressed in the tested condition compared to the control, whereas it is under-expressed when a FC is negative. Du column: D indicates the presence of a duplication: two different probes are tagging the same gene. Long intergenic non-coding RNAs data are displayed with a blue filling and small nucleolar RNAs data are displayed with a pink filling. Probes differentially expressed for both concentrations (10 nM and 10 μM) are displayed with a yellow filling.
(XLSX)

S4 Table Differential analysis of housekeeping genes for the three bisphenols (A, F and S) at both concentrations (10 nM and 10 μM). The significant log_2_ fold changes along with the p-values are displayed. NS stands for non-significant. The highlighted housekeeping genes (pink background) show no differential expression in any condition compared to the control.
(XLSX)

S5 Table Taqman probes (mRNA and mirRNA) used in the qPCR. (XLSX)

S6 Table Comparison of the microarray analysis with the qPCR analysis. The ten genes that were differentially expressed in the microarray analysis for the three bisphenols (A, F and S) at both concentrations (10 nM and 10 μM) were analyzed. The log_2_ fold changes (positive when the probe is over-expressed; negative when the probe is under-expressed), along with the p-values are displayed for each technology.
(XLSX)
