# Supplementary figures and images for: Low-dose exposure to bisphenols A, F and S of human primary adipocyte impacts coding and non-coding RNA profiles
Source: PLoS One. 2017 Jun 19;12(6):e0179583. doi: 10.1371/journal.pone.0179583 (PMC5476258; doi:10.1371/journal.pone.0179583)

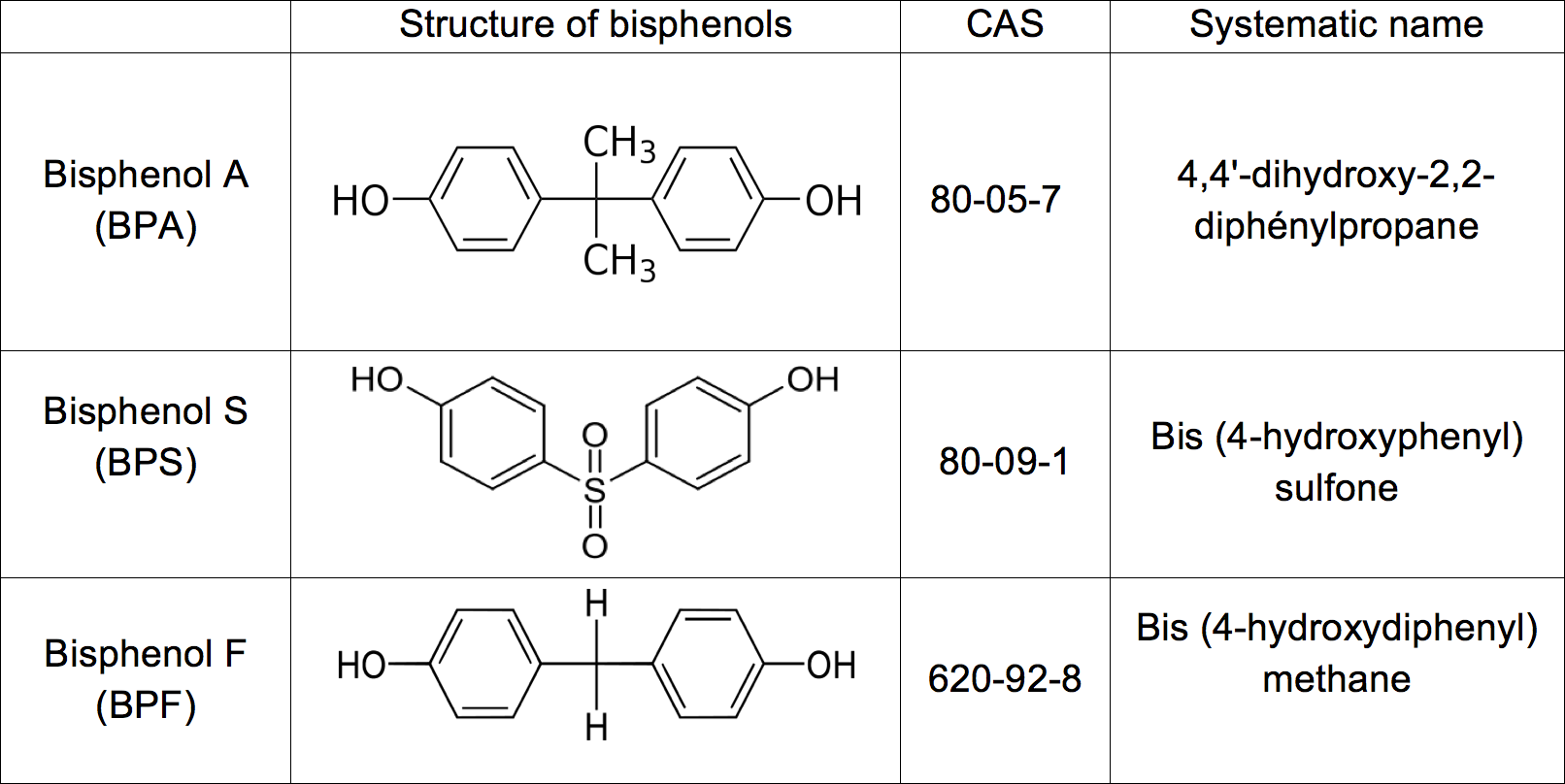

Supplement: S1 Fig — (TIF) [file pone.0179583.s001.tif]

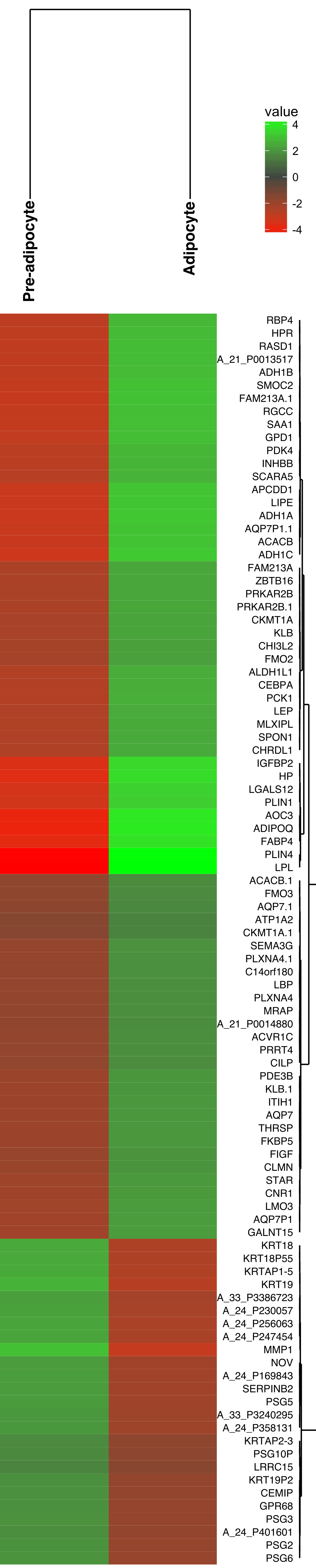

Supplement: S2 Fig — The difference between the average expression in the pre-adipocyte and the adipocyte with the global expression is represented. (TIF) [file pone.0179583.s002.tif]

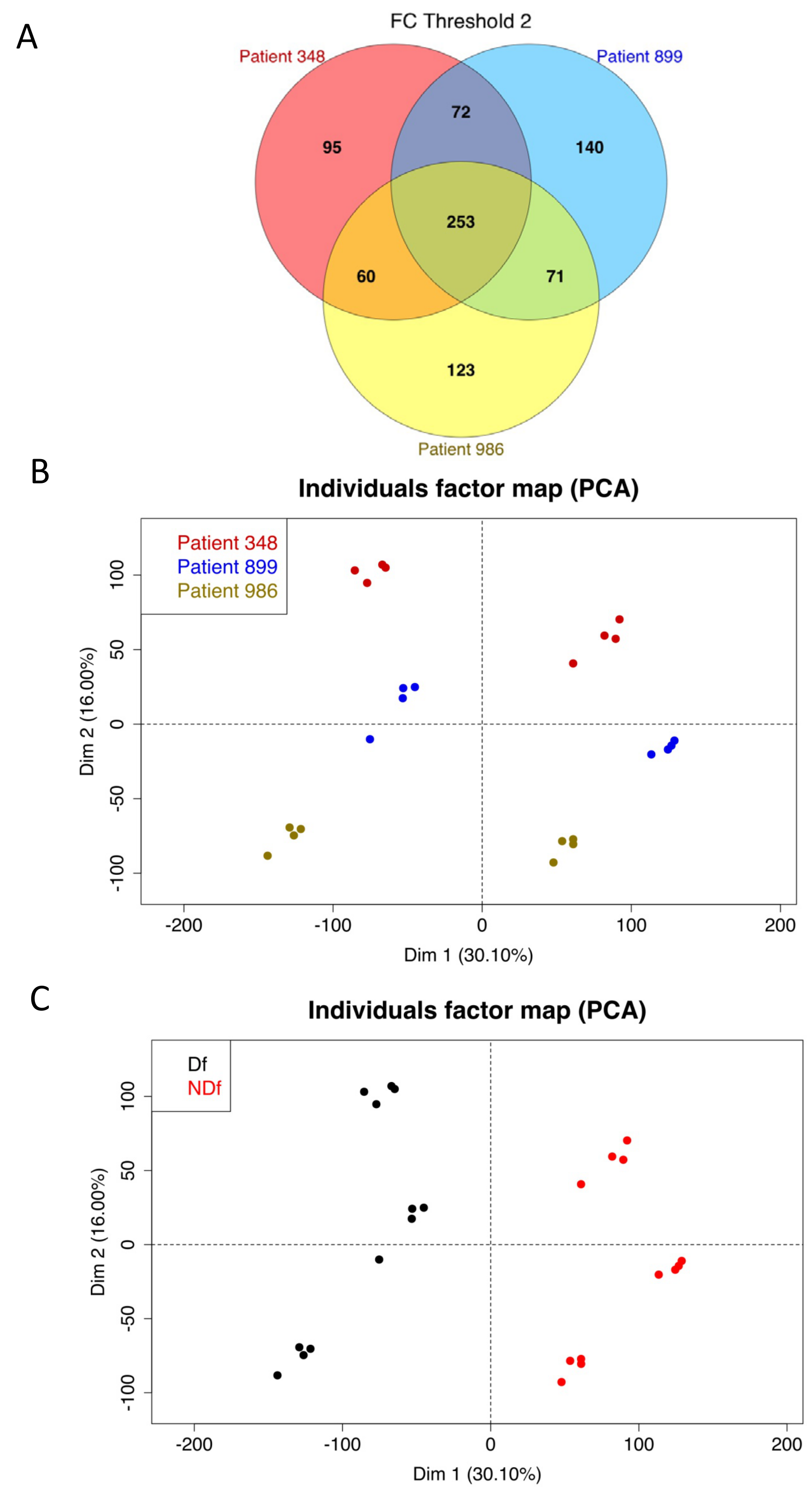

Supplement: S3 Fig — A) Venn diagram of differentially expressed probes for each patient before and after ten days of differentiation, a probe is considered as differentially expressed if log2FC>2. B) Individual representation of the first two axes of the principal component analysis on all probes for each patient before and after ten days of differentiation (colored according to patient). C) Individual representation of the first two axes of the principal component analysis on all probes for each patient before and after ten days of differentiation (colored according to differentiation stage). (TIF) [file pone.0179583.s003.tif]

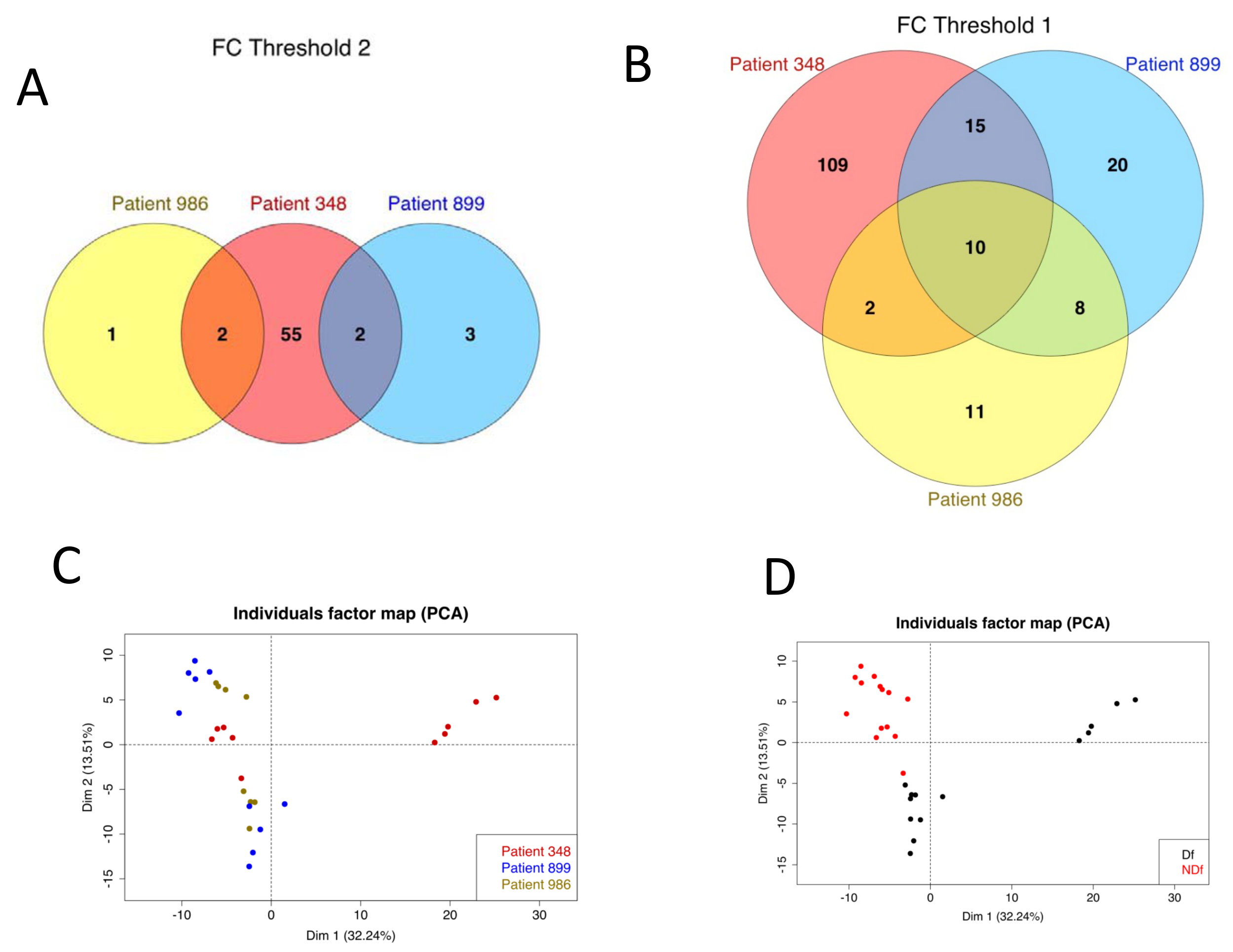

Supplement: S4 Fig — A) Venn diagram of differentially expressed probes for each patient before and after ten days of differentiation, a probe is considered as differentially expressed if log2FC>2. B) Venn diagram of differentially expressed probes for each patient before and after ten days of differentiation, a probe is considered as differentially expressed if log2FC>1. C) Individual representation of the first two axes of the principal component analysis on all probes for each patient before and after ten days of differentiation (colored according to patient). D) Individual representation of the first two axes of the principal component analysis on all probes for each patient before and after ten days of differentiation (colored according to differentiation stage). (TIF) [file pone.0179583.s004.tif]
